# Supplementary material for: Predictors of Maternal Serum Concentrations for Selected Persistent Organic Pollutants (POPs) in Pregnant Women and Associations with Birth Outcomes: A Cross-Sectional Study from Southern Malawi
Source: Int J Environ Res Public Health. 2023 Mar 28;20(7):5289. doi: 10.3390/ijerph20075289 (PMC10093902; doi:10.3390/ijerph20075289)
Supplement: Supplementary file 1 [file ijerph-20-05289-s001.zip › Supplementary Table S1_Method detection limits (MDL).pdf]

Supplementary Table S1: Method detection limits (MDL) concentrations (wet weight pg/mL) for all POPs analysed at the laboratory.

POPs	Mean MDL Concentrations	Median MDL Concentrations	Min - Max MDL Concentrations
o,p′-DDE	0.3	0.3	0.07 to 1.53
p,p′-DDE	0.4	0.3	0.09 to 4.26
o,p′-DDD	0.6	0.5	0.20 to 1.44
p,p′-DDD	1.8	1.5	0.34 to 7.14
o,p′-DDT	0.7	0.6	0.09to 2.99
p,p′-DDT	2.0	1.8	0.46 to 6.73
HCB	1.1	0.9	0.02 to 12.74
Mirex	6.2	5.8	0.76 to 23.85
a-HCH	2.1	1.8	0.85 to 9.67
g-HCH	2.1	2.0	0.79 to 7.59
b-HCH	3.6	3.5	1.69 to 7.85
Heptachlor	0.3	0.2	0.002 to 2.89
t-CD	0.5	0.3	0.01 to 8.51
c-CD	0.5	0.3	0.02 to 8.37
t-NC	1.8	0.6	0.02 to 24.44
cis-NC	0.9	0.3	0.02 to 28.94
Oxychlordane	6.6	5.9	0.05 to 35.33
cis-Heptachlorepoxide	2.6	2.3	0.06 to 44.24
Dieldrin	6.3	5.1	0.07 to 41.35
