# Supplementary material for: Predictors of Maternal Serum Concentrations for Selected Persistent Organic Pollutants (POPs) in Pregnant Women and Associations with Birth Outcomes: A Cross-Sectional Study from Southern Malawi
Source: Int J Environ Res Public Health. 2023 Mar 28;20(7):5289. doi: 10.3390/ijerph20075289 (PMC10093902; doi:10.3390/ijerph20075289)
Supplement: Supplementary file 1 [file ijerph-20-05289-s001.zip › Supplementary Table S2_Socio-demographic characteristics .pdf]

Supplementary Table S2: Socio-demographic characteristics of recruited women and their neonates

Variable	Characteristic	Total	Place of residence		p-value	Missing data (count)
			Urban	Rural		
Total participants (n)		605	308	297		
Age (Years)	Mean (SD)	24.8 (6.22)	25.6 (5.68)	23.9 (6.67)	<0.001 [a]	1
Mode of delivery (%)	CS	91 (16.0)	53 (18.6)	38 (13.4)	0.111 [b]	37
	SVD	474 (83.5)	229 (80.4)	245(86.6)		
	Breech	2 (0.4)	2 (0.7)	0 (0.0)		
	Vac extr	1 (0.2)	1 (0.4)	0 (0.0)		
Gravidity (%)	1	220 (36.4)	78 (25.3)	142 (47.7)	<0.001 [b]	0
	2	157 (25.9)	106 (34.4)	51 (17.1)		
	3	117 (19.3)	73 (23.8)	44 (14.8)		
	4	111 (18.4)	51 (16.5)	60 (20.4)		
Parity (%)	0	207 (34.5)	61 (20.2)	146 (49.2)	<0.001 [b]	6
	1	137 (22.8)	86 (28.5)	51 (17.2)		
	2	256 (42.7)	155 (51.3)	100 (33.6)		
Maternal education (%)	None / primary	325 (53.9)	118 (38.6)	207 (69.7)	<0.001 [b]	2
	Secondary/ Tertiary	278 (46.1)	188 (61.4)	90 (30.3)		
Marital Status (%)	Married	544 (90.0)	283 (91.9)	260 (87.6)	0.081 [b]	0
	Single	61 (10.0)	25 (8.1)	37 (12.4)		
Breast Feeding (%)	Yes	355 (59.1)	208 (68.0)	147 (49.8)	<0.001 [b]	4
	No	246 (40.9)	98 (32.0)	148 (50.2)		
Drinking water source (%)	Tap	322 (53.5)	298 (96.8)	24 (8.2)	<0.001 [b]	3
	Lake/ s-well	137 (22.8)	2 (0.7)	135 (45.9)		
	Borehole	143 (23.8)	8 (2.6)	135 (45.9)		
Pesticides use at home (%)	Yes	124 (20.5)	20 (6.5)	104 (35.1)	<0.001 [b]	1
	No	480 (79.5)	288 (93.5)	192 (64.9)		
Fishing (%)	Yes	9 (1.5)	0 (0.0)	9 (3.0)	0.002 [b]	0
	No	596 (98.5)	308 (100.0)	288 (97.0)		
Gestational age (weeks)	Mean (SD)	37.58 (1.53)	37.47(1.43)	37.71(1.62)	0.075 [a]	64
Birth weight (Kg)	Mean (SD)	3.09 (0.47)	3.18 (0.46)	3.00(0.46)	<0.001 [a]	33
Birth length (cm)	Mean (SD)	45.04 (4.28)	45.66(5.55)	44.51(2.65)	0.002 [a]	64
Head circumference (cm)	Mean (SD)	33.17 (1.83)	33.14 (1.94)	33.19(1.72)	0.845 [a]	64

SD: standard deviation of mean

[a] Mann–Whitney U test (urban vs rural).

[b]Chi-square test (urban vs rural).
